# Endobronchial Hamartoma as a Rare Cause of Recurrent Respiratory Symptoms: Case Report and Literature Review

**DOI:** 10.7759/cureus.5489

**Published:** 2019-08-26

**Authors:** Artem Minalyan, Neethu Gopisetti, Adrian Estepa, Harshwant Grover, Rajeshkumar Patel

**Affiliations:** 1 Internal Medicine, Abington Jefferson Health, Abington, USA; 2 Pulmonary and Critical Care Medicine, Abington Jefferson Health, Abington, USA

**Keywords:** hamartoma, endobronchial hamartoma, lung mass, hemoptysis, benign tumor

## Abstract

Most of the endobronchial lesions are malignant in origin. In rare instances, benign lesions occupying the endobronchial tree can mimic malignant neoplasms on conventional imaging tests. We present a case of a middle-aged male patient who was admitted to our hospital with recurrent hemoptysis concerning for lung cancer on computed tomography (CT) of the chest. Biopsy of the mass obtained via bronchoscopy revealed a benign lesion most consistent with lipomatous hamartoma, which is known to constitute only 10% of all pulmonary hamartomas. We also present the data of a comprehensive literature review of the epidemiology, clinical symptoms, diagnosis, treatment, and prognosis of patients with endobronchial hamartomas.

## Introduction

Pulmonary hamartomas are the most common benign lung tumors [1]. It is estimated that only 10% of pulmonary hamartomas are found in the endobronchial tree [2]. They typically occur between the fifth and seventh decades of life. The manifestations of pulmonary hamartomas depend on the location of the tumor. In fact, most pulmonary hamartomas have an asymptomatic course and can be detected incidentally on chest imaging. However, endobronchially located hamartomas can cause symptoms related to endobronchial obstruction: recurrent respiratory tract infections, dyspnea, cough, and hemoptysis.

## Case presentation

 A 49-year-old male of Mediterranean origin without a significant past medical history presented to the emergency department for evaluation of hemoptysis and shortness of breath. He stated that his symptoms started one day ago. He denied fevers, chills, chest pain, and purulent cough. There was no weight loss, hematemesis, hematochezia, rash, or mucosal bleeding. He denied a history of recent travel and had no history of tuberculosis exposure. He is a former smoker (three pack-years; quit 18 years ago). He drank alcohol socially and worked at a local restaurant.

**Figure 1:**
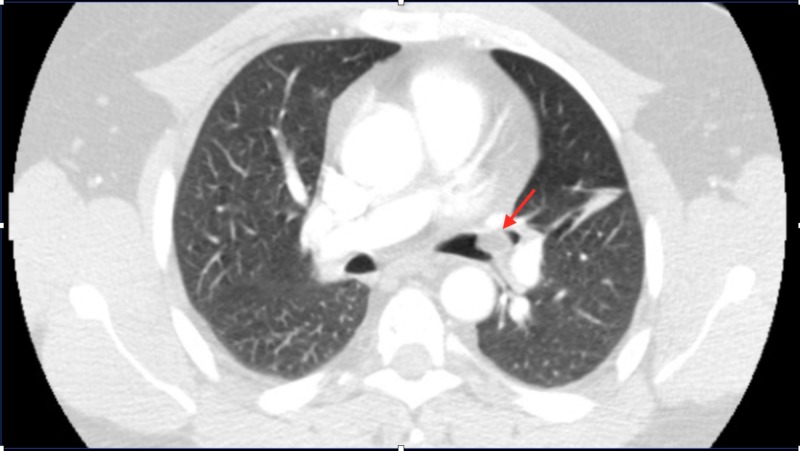
Computed tomography of the chest with IV contrast Endobronchial mass in the left upper lobe (LUL) bronchus (red arrow) resulting in subsegmental atelectasis is noted. IV: intravenous

Upon further questioning, the patient admitted to having similar symptoms about seven months ago. At that time, he was seen by his primary care physician, diagnosed with bronchitis, and treated with antibiotics. No recurrence of symptoms was noted prior to the current presentation. Vital signs, as well as physical findings, were unremarkable. The complete blood count, metabolic panel, and coagulation profile were normal. Chest X-ray showed decreased lung volumes and a very small lingular infiltrate versus atelectasis. Computed tomography (CT) of the chest with intravenous contrast revealed a 14-mm endobronchial mass in the left upper lobe (LUL) bronchus, resulting in subsegmental atelectasis of the lingular segment (Figure 1).

**Figure 2:**
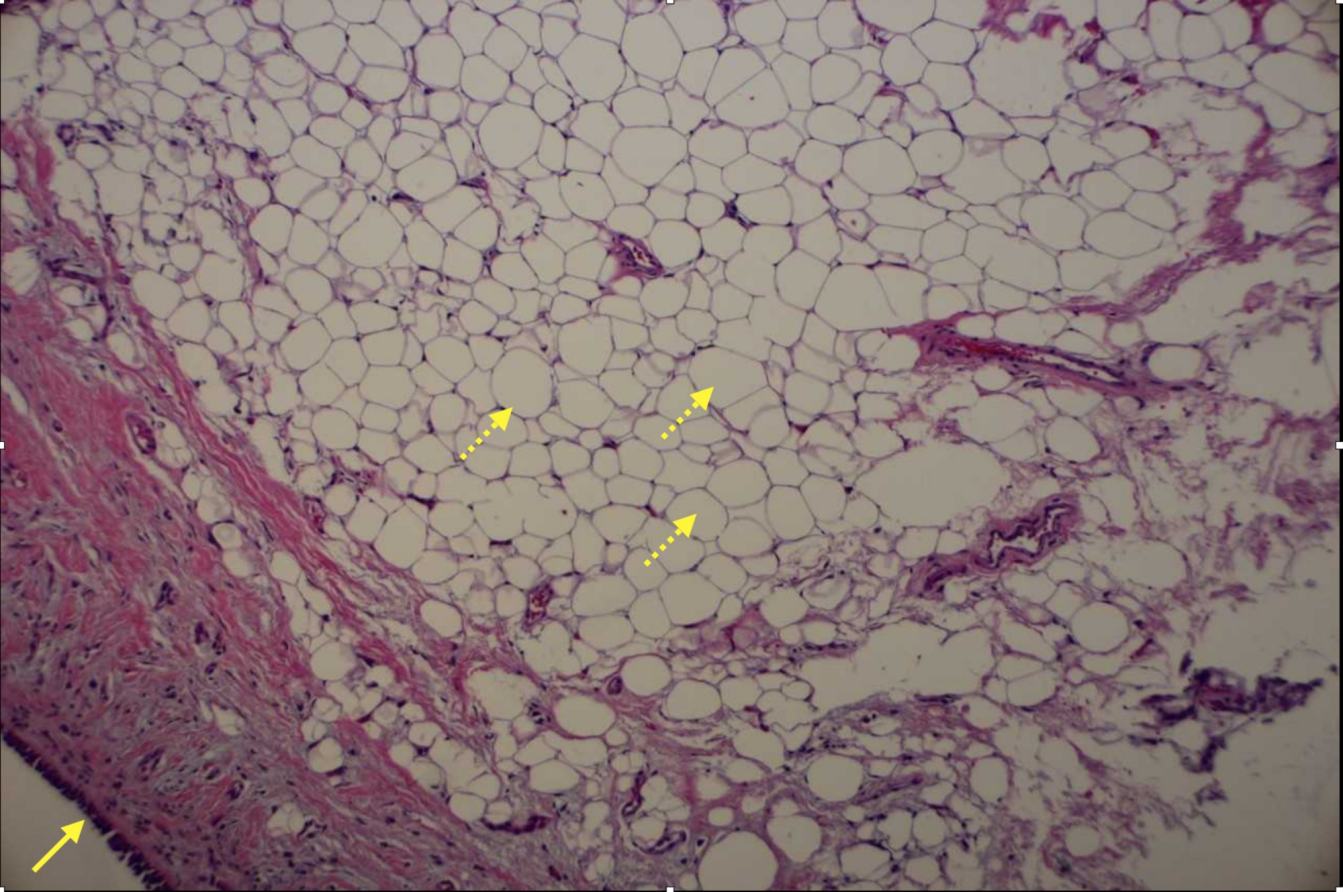
Endobronchial mass biopsy (stained with hematoxylin and eosin) There is an endobronchial epithelium on the surface (solid arrow). A nodular proliferation of mature adipose tissue can be seen (dashed arrow).

On Day 2, the patient underwent bronchoscopy. In the distal left mainstem bronchus, a white mass was seen growing out of the LUL bronchus at LC1, which was completely occluding the orifice with an inability to visualize LUL or lingular bronchi. Multiple biopsies were done with debulking of the mass and subsequent cauterization. An endobronchial ultrasound (EBUS)-guided transbronchial needle aspiration (TBNA) of an adjacent lymph node was performed as well. The patient was discharged on Day 3 and recommended to follow up with pulmonology. The endobronchial biopsy showed a lipomatous hamartoma (Figure 2).

Bronchial washings, as well as fine-needle aspiration cytology (FNAC), were negative for malignant cells. One month after discharge, the patient continued to complain of shortness of breath, and no hemoptysis was reported. It was decided to proceed with repeat bronchoscopy. No bronchial obstruction was noted. Biopsy at the previous site was obtained, which showed only focal fibrosis with giant cell reaction. His symptoms eventually resolved, and he continues to follow-up regularly with pulmonology.

## Discussion

The term “hamartoma” was originally introduced by Albrecht in 1904. It is derived from the Greek “hamartia” (defect) and “oma” (tumor) [3]. A hamartoma is defined as a benign malformation of an abnormal mixture of normal cells and tissues that belong to the same organ. It usually contains mesenchymal and epithelial elements. The distinctive feature of a hamartoma is the presence of at least two mesenchymal elements (for instance, cartilage and fat). Hamartomas are most commonly present at birth, however, they may develop in adulthood.

Pulmonary hamartoma is the third most common cause of pulmonary nodules [4]. It is also considered the most common benign lung tumor. Depending on the location, there are two main variants: (1) peripheral (greater than 90% of cases) and (2) endobronchial. Pulmonary hamartomas, in general, are more common in middle age. Most of the hamartomas are asymptomatic and discovered incidentally on chest X-ray or computed tomography. However, those that are greater in size or located inside the bronchi may present with a variety of symptoms caused mainly by compression or obstruction [5]. Clinical symptoms include coughing, wheezing, and intermittent shortness of breath, leading to the misdiagnosis of asthma or chronic obstructive pulmonary disease. Similar to any pulmonary nodule, obtaining a tissue sample is required for confirming the diagnosis of pulmonary hamartoma. After the diagnosis is established, several therapeutic interventions can be suggested depending on the size, location (peripheral vs endobronchial), and symptoms of the patient. They include both endobronchial approach (resection, ablation, electrocauterization, etc.) and lung surgery (resection, lobectomy, pneumonectomy, bronchotomy, etc.) [6-7].

Endobronchial hamartoma, although considered the most common endobronchial benign neoplasm, is known to be a very rare cause of an endobronchial mass. Notably, most of the endobronchial tumors are malignant. Interestingly, all benign endobronchial tumors combined together constitute about 2% of all lung tumors [8].

We performed a comprehensive literature search in PubMed/Medline to find all the published case reports and case series of patients with endobronchial hamartoma from inception till December 20, 2018. We used the following terms: “endobronchial,” “pulmonary,” and “hamartoma.” The Boolean operators “OR” (“endobronchial,” “pulmonary”) and “AND” were selected to specify the search outcome. All terms were searched using the “Abstract/Title” field. We only included cases of adult patients. Only original case reports and case series were included for further review. Cases that did not have abstracts and were published elsewhere were excluded. We were able to identify 80 articles that met the inclusion criteria [9]. Overall, 217 patients were reported. Several variables were further reviewed: age, gender, race, symptoms, duration of symptoms, history of smoking, history of cancer, location of the tumor, treatment, and follow-up. Predictably, some variables were not available in several reported cases. The average age (in years) of patients affected with endobronchial hamartoma was 56 (ranging from 23 to 97). It is reportedly more common in Caucasian males and is relatively rare in African American females. The symptoms most commonly described by patients include productive cough and shortness of breath. The duration of symptoms before the diagnosis was made was found to be extremely variable (ranging from five days to 15 years). Interestingly, a significant proportion of non-smokers without any history of malignancy were also affected. Out of 102 cases that reported the localization of the tumor, 53 patients had a tumor in the left bronchial tree and 46 patients in the right one. Only three patients were known to have tracheal involvement [10-12]. Tsitouridis et al. reported a case of an endobronchial (left main bronchus) lipomatous hamartoma with an extra-bronchial extension into the mediastinum [13]. In most cases, the diagnosis was made via obtaining tissue biopsy during diagnostic bronchoscopy. Unfortunately, interpreting endobronchial biopsy results may be challenging and, therefore, warrants pursuing more aggressive intervention. For instance, Poyrazoglu et al. reported a case of an endobronchial hamartoma occupying the right upper lobe bronchus. The initial biopsy obtained via bronchoscopy showed chronic non-specific inflammation. The results of a repeat biopsy were consistent with squamous metaplasia, which alerted the medical team to perform a lobectomy [14]. Overall, the therapeutic strategy offered to patients with endobronchial hamartomas vary, and include a wide range of bronchoscopic interventions (clamping, cauterization (both electro- and argon plasma), excision, debulking) and surgical ones (resection, lobectomy, pneumonectomy, tracheotomy, bronchotomy, etc.). During follow-up (2 months - 2 years), the majority of patients did not demonstrate signs or symptoms of recurrence.

## Conclusions

Despite the fact that pulmonary hamartoma is the most common type of benign lung tumor, its endobronchial localization is known to be rare. In contrast to the peripheral subtype, endobronchial hamartomas usually manifest with recurrent respiratory symptoms. Chest X-ray may be unremarkable in patients with endobronchial hamartoma, therefore, more advanced diagnostic tests are required for further work-up. CT of the chest can usually detect a low-attenuation endobronchial mass. Diagnostic and therapeutic bronchoscopy is the test of choice when an endobronchial mass is suspected. Of note, in rare instances, endobronchial biopsy findings may reveal metaplastic changes that can present a diagnostic challenge. Endoscopic intervention is a safe and effective therapeutic option for selected cases. The prognosis of endobronchial hamartoma is generally favorable. There is no consensus regarding the follow-up interval and its frequency. Once removed, the chances of the recurrence of endobronchial hamartoma are very low.
